# ConMLP: MLP-Based Self-Supervised Contrastive Learning for Skeleton Data Analysis and Action Recognition

**DOI:** 10.3390/s23052452

**Published:** 2023-02-22

**Authors:** Chuan Dai, Yajuan Wei, Zhijie Xu, Minsi Chen, Ying Liu, Jiulun Fan

**Affiliations:** 1 School of Computing and Engineering, University of Huddersfield, Huddersfield HD1 3DH, UK; 2 School of Cyberspace Security, Xi’an University of Posts and Telecommunications, Xi’an 710061, China; 3 International Joint Research Center for Wireless Communication and Information Processing, Xi’an 710121, China; 4 School of Communications and Information Engineering, Xi’an University of Posts and Telecommunications, Xi’an 710061, China

**Keywords:** human action recognition, skeleton data, multi-layer perceptron, self-supervised learning

## Abstract

Human action recognition has drawn significant attention because of its importance in computer vision-based applications. Action recognition based on skeleton sequences has rapidly advanced in the last decade. Conventional deep learning-based approaches are based on extracting skeleton sequences through convolutional operations. Most of these architectures are implemented by learning spatial and temporal features through multiple streams. These studies have enlightened the action recognition endeavor from various algorithmic angles. However, three common issues are observed: (1) The models are usually complicated; therefore, they have a correspondingly higher computational complexity. (2) For supervised learning models, the reliance on labels during training is always a drawback. (3) Implementing large models is not beneficial to real-time applications. To address the above issues, in this paper, we propose a multi-layer perceptron (MLP)-based self-supervised learning framework with a contrastive learning loss function (ConMLP). ConMLP does not require a massive computational setup; it can effectively reduce the consumption of computational resources. Compared with supervised learning frameworks, ConMLP is friendly to the huge amount of unlabeled training data. In addition, it has low requirements for system configuration and is more conducive to being embedded in real-world applications. Extensive experiments show that ConMLP achieves the top one inference result of 96.9% on the NTU RGB+D dataset. This accuracy is higher than the state-of-the-art self-supervised learning method. Meanwhile, ConMLP is also evaluated in a supervised learning manner, which has achieved comparable performance to the state of the art of recognition accuracy.

## 1. Introduction

The RGB videos provided by traditional datasets contain rich semantic information, which can be extracted for action recognition and classification, which are fundamental problems in computer vision. However, they also bring various forms of noise. For example, backgrounds unrelated to the actions, poor illumination, and object occlusions [1]. On the other hand, other video representations have emerged due to the development of video capture technologies. Skeleton data generated by depth sensors [2] are highly valuable modalities. They have a low amount of data and take fewer computing resources. Moreover, they are affected relatively little by interference factors, such as illumination and background [1].

Therefore, studies on recognizing and classifying actions through skeleton-based data have been extensively explored. Most of these studies are derived from image-based recognition [3,4]. Convolutional neural network (CNN)-based approaches express skeleton joint coordinates across multiple frames in terms of pseudo images, presenting this as an image processing challenge [3]. As recurrent neural network (RNN)-based methods, skeletons are encoded into a sequence of structured coordinate vectors. They exploit RNNs’ ability to deal with time-series data [5], while graph convolutional network (GCN)-based approaches combine the hierarchical features of skeleton data to represent frame sequences as connections and updates in the interrelationships between graph vertices and edges [6,7].

Although a remarkable effect is achieved, several issues are observed: (1) Complex models can be quite expensive in terms of computational resources. The FLOPs (FLoating-point OPerations) of these models tend to be several orders of magnitude larger than those of naive architectures. (2) For supervised learning approaches, the need for massively labeled data is always a disadvantage. (3) The combination of complex algorithms and large models is difficult to truly deploy into production applications.

In contrast, ConMLP, which is proposed in this paper in conjunction with recent advances in MLP, is a simple framework. It does not contain any computationally expensive layers such as convolution operations or attention mechanisms. It is built on a naive MLP as a base encoder network combined with a contrastive loss function, which does not fall into the categories of CNN-, RNN-, or GCN-based approaches. It can be applied to both self-supervised learning and supervised learning. The inference results of ConMLP have a top one of 96.9% on the NTU RGB+D dataset. This accuracy is 0.2% higher than the state-of-the-art self-supervised learning approach [8], and it is on par with the best result of supervised learning [9]. The code of this research is available at: https://github.com/ChuanDai/ConMLP (accessed on 28 December 2022).

Our main contributions are summarized below:

1. A novel MLP-based self-supervised learning framework is proposed, which significantly reduces computational complexity while achieving state-of-the-art performance. It saves more than 95% of FLOPs compared with the ResNet50-based encoder network;
2. The generic contrastive learning metrics of image classification [10] have been transformed to action recognition on skeleton sequences, which demonstrated its flexibility and feasibility for wider applications;
3. For validating the devised framework, ConMLP is also evaluated against benchmarking supervised learning approaches with superior performances recorded.

## 2. Related Work

### 2.1. Visual Representation Learning

Visual representation learning methods generally fall into the following three categories: handcrafted pretext tasks, pixel-level generation, and contrastive learning.

#### 2.1.1. Handcrafted Pretext Tasks

To provide reasonable and efficient representations for downstream tasks, such methods need to use pretext tasks for pre-training. However, the formation of such pretext tasks is usually based on heuristic inspiration [11,12]. Even if good results can be reached by a larger network and a long training time, such designs typically have poor generalization [13].

#### 2.1.2. Pixel-Level Generation

In general, adversarial models are used for pixel-level generation. The primary goal of adversarial models is to reproduce data distribution as effectively as is feasible to help recognize objects [14]. The main criticism of this kind of method is the intensive computational burden; additionally, whether it is necessary for representation learning is also worth discussing [15].

#### 2.1.3. Contrastive Learning

Compared with the above two kinds of methods, contrastive learning approaches achieve the best performance, and their interpretability is also recognized [10]. The architecture of combining a base encoder network and a projection network can be supported by the theoretical basis of such methods [14]. Triplet [16] is a supervised contrastive-learning-based model. Apart from the anchor, each mini-batch contains only one positive and one negative sample. This means only one negative sample was compared in a mini-batch, while other classes of negatives were ignored. Meanwhile, it was necessary to exploit hard-negative mining, which was computationally expensive. N-pair [17] is an extended version of Triplet loss. All negative samples in a mini-batch participated in the computation, and more reasonable representations were learned. However, the number of positive samples does not change. SimCLR [18] is characterized by self-supervised loss with an optimized combination of schemes, demonstrating the importance of augmented data. Positive samples are generated by data augmentations, while negative samples are made up of the remaining 2(*N* − 1) samples in two copies of a mini-batch. It can achieve much higher performance than Triplet, even if not using hard-negative mining. Additionally, SupCon [14] extended the loss proposed in SimCLR to also adapt supervised contrastive learning.

### 2.2. Skeleton-Based Action Recognition

#### 2.2.1. Self-Supervised Methods

VaRe [8] is a GCN-based framework combined with a view-normalization generative adversarial network (VN-GAN) and subject-independent network (SINet). This framework could recognize actions without the knowledge of view- and subject-specific habits. SRCL [19] consisted of two networks, an online and a target network, and used the distribution of scores of inter-instance similarity as a relational metric to introduce relational consistency learning. MG-AL [20] treated the self-supervised for action representation learning as a self-attention problem, and it did not involve any data augmentations. CRRL [21] included a two-stage architecture for learning and representation fusing. Additionally, a new data augmentation scheme called velocity was proposed. In [22], a framework that combined the attention mechanism and contrastive learning was proposed. Although no GCN structures were employed, data augmentation still played a critical role. SKT [23] is also characterized by a contrastive learning strategy. It used a Barlow Twins objective function to minimize the agreement between similar samples without negative samples being required. GLTA-GCN [24] is a self-attention-equipped framework with intensive GCN structures. It introduced two losses to serve the framework of a multi-task process. SEMN [25] introduced a skeleton modality called skeleton edge motion and has a loss function to help perform self-supervised learning. In [26], the authors introduced a loss to estimate noise by contrastive learning and used several spatial–temporal based data augmentation schemes. MCAE [27] utilizes the modeling process as two levels, namely a lower level and a higher level for dividing and aggregating the spatial–temporal signal, respectively.

Most of these methods chose GCN as the backbone network [8,19,20,23,24,26]. On the other hand, only a few methods did not employ contrastive loss functions [8,20,25]. This shows that the idea of using contrastive learning to tackle self-supervised learning is mainstream thinking. Moreover, only a few methods did not use data augmentations [20,24,25], which also shows that it is indispensable for contrastive learning. The proposed ConMLP in this paper is a learning framework that combines a contrastive function as the loss with generating pairs of data through augmentation.

In addition to action recognition on skeleton data, self-supervised learning is also applied in several real-world tasks, such as in facial landmark analysis [28,29] and digital healthcare [30].

#### 2.2.2. Supervised Methods

ST-GCN [6] is a spatial–temporal method to tackle human action recognition with GCN, which laid a foundation for subsequent studies. Moreover, 2s-AGCN [31] was proposed to address the limitations of ST-GCN, including its ability to only deal with first-order information. Instead, bone information including length and direction was incorporated in addition to the joint information. Additionally, a data-driven approach was used to parameterize both the global graph and individual graph to extend the flexibility of the model. MS-G3D [7] was a feature extractor by fusing a disentangled multi-scale aggregation scheme and a unified spatial–temporal graph convolution (G3D) operator, and dilated convolutions [32] were applied to multi-scale aggregation, which effectively controlled the complexity of the network architecture. MST-GCN [33] is a multi-scale spatial graph convolution (MS-GC) module combined with a multi-scale temporal graph convolution (MT-GC) module that models distance joints relations and long-range temporal information. In addition, both MS-G3D and MST-GCN considerably increased temporal receptive fields, but they were adopted in different ways. MS-G3D utilized paralleled 3 × 1 kernel sizes combined with dilated windows, while MST-GCN used a single block of a hierarchical architecture.

Conventional GCN-based methods often have high computational complexity. To reduce the computational burden of GCN, Shift-GCN [34], which was inspired by shift convolution [35], exploited the lightweight shift graph operations to provide flexible receptive fields for both spatial graphs and temporal graphs. However, the proposed ConMLP does not contain any GCN structure. While it achieves state-of-the-art of performance, the computational complexity is substantially reduced, even compared with Shift-GCN.

### 2.3. Multi-Layer Perceptron

#### 2.3.1. Advances in MLP Architecture

Multi-layer perceptron (MLP)-based models have recently received a lot of attention. MLP-Mixer [36] showed that even without using CNN and self-attention, the model based on MLP could also achieve excellent performance in image classification. MLP-Mixer relied solely on an MLP that was repeatedly implemented in the spatial domain or feature channels, as well as matrix multiplication, data scaling, and nonlinear layers. This was much more efficient in terms of computational complexity.

ResMLP [37] adopted a similar approach to MLP-Mixer, using two MLPs acting on different directions of image patches. Its strength was that ResMLP could rapidly train a high-performing model on a smaller ImageNet branch. gMLP [38] introduced a spatial gating unit into the model without using self-attention. It evaluated performance on both computer vision and natural language processing. Compared with MLP-Mixer, it reduced parameters by 66% while improving the performance by 3%. These models were similar in structure but differ in block design details.

Without blindly emphasizing the importance of MLP architecture, RepMLP [39] and CycleMLP [40] were attempts to combine MLP with CNNs or self-attention mechanisms.

#### 2.3.2. MLP’s Resurgence

There are many reasons for the remarkable performance of MLPs, including the increase in computing power and datasets improvements. Moreover, modern MLP models have many commonalities in their implementation. For example, most of them applied Gelu [41] as the activation function [36,37,38,40], and layer normalization [42] was exploited instead of batch normalization [36,37]. Additionally, adding skip connections [43] was a common method while extending more layers [36,37].

Our paper follows these practices, using Gelu and layer normalization. The skip connection will be investigated in future work. In Section 4.6, the role played by the number of MLP hidden layers is also explored.

## 3. ConMLP Framework Design

Assume that an input skeleton sequence can be represented as $x=\left(x_{1},x_{2},\ldots ,x_{T}\right)$ containing *T* consecutive skeleton frames, where $x_{i}\in {\mathbb{R}}^{S\times J\times 3}$ contains coordinates of *S* subjects with *J* different 3D body joints. The training dataset $\Phi ={\left\{x^{i}\right\}}_{i=1}^{N}$ consists of *N* skeleton sequences from different actions, and their views and subjects may be different. Each skeleton sequence $x^{i}$ corresponds to a label $y_{i}$, where $y_{i}\in \left\{a_{1},a_{2},\ldots ,a_{c}\right\}$, $a_{i}$ denotes $i^{th}$ action class, and *c* is the number of classes. The goal is to learn a set of valid representations by $x^{i}$ without introducing any labels.

**Figure 1:**
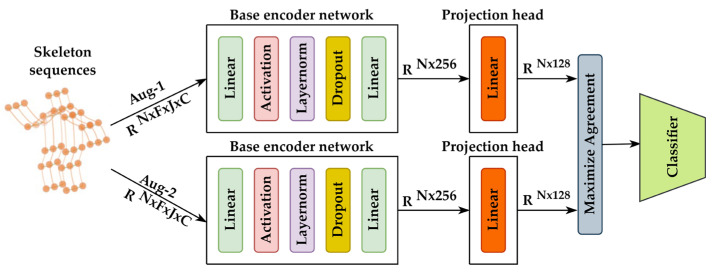
An overlook of the ConMLP framework. *N* denotes the mini-batch size; *F* denotes the number of frames in a skeleton sequence; *J* denotes the number of joints; *C* denotes the 3D coordinates.

This paper adopts a widely used evaluation metric [10,18]. First, data augmentations are applied to the input mini-batch data. Two copies of mini-batch data enhanced by augmentations are forward propagated through the base encoder network. Then, the representations of the output are further trained by a projection head, which is not used in the inference stage. Next, the contrastive loss is calculated based on the output of the projection network. Finally, a linear classifier is trained on top of them, while the representations are frozen (Figure 1).

### 3.1. Data Augmentation Schemes

For self-supervised contrastive learning, data augmentation plays a crucial role. Positive samples of the same class as the anchor are generated through data augmentation, and the selection of an augmentation scheme can directly affect the quality of representation learning [18]. For each piece of the input skeleton sequence, i.e., each action, two sets of augmented data are generated, each representing a varied view of the skeleton sequence.

Different from the augmentation schemes for images, skeleton-based data augmentation mainly includes shear, reverse, rotation, joint mask, Gaussian blur, Gaussian noise, channel mask, etc. Shear and reverse are adopted in this paper for optimal performance [44,45].

Shear transformation is one of the spatial linear transformations. In the context of an image, it can be interpreted as stretching either side of a rectangle, eventually turning it into a parallelogram. However, for skeleton data, each joint is projected in a predefined direction, so that a skeleton frame is projected to a certain viewpoint. A generic 3D shear transformation matrix can be used to generate sheared sequences for skeleton data. The shear transformation matrix can be defined as:

$$S=\left(\begin{array}{ccc} 1 & S_{X}^{Y} & S_{X}^{Z}\\ S_{Y}^{X} & 1 & S_{Y}^{Z}\\ S_{Z}^{X} & S_{Z}^{Y} & 1 \end{array}\right)$$

where $S_{X}^{Y},S_{X}^{Z},S_{Y}^{X},S_{Y}^{Z},S_{Z}^{X},S_{Z}^{Y}\in \left[-1,1\right]$ are randomly generated shear factors, which control the amplitude of transformation from one dimension to another. The joints coordinate of the original skeleton sequence can all be transformed by this matrix.

Reverse refers to reversing the view of the temporal order. For NTU RGB+D datasets with (*N*, *F*, *J*, *C*) structures, the reverse is exploited for the second dimension, i.e., frames. One skeleton sequence has a 50% chance of being reversed. Nevertheless, the reverse is one of the best-proven augmentations for skeleton sequences.

### 3.2. Base Encoder Network

The base encoder network is to transform input data into representation vectors. The two groups of augmented data are forward propagated through the base encoder network, with a pair of representation vectors being generated.

In this paper, a naive MLP with 256 hidden layers is employed as the base encoder network. Following a common MLP design principle, the base encoder consists of linear layers followed by activation, normalization, dropout, and linear layers (Figure 1). Gelu [41] is applied for activation. For normalization, layernorm [42] is used for training stability instead of applying batch normalization. As a convention, dropout is used to avoid overfitting.

Moreover, a ResNet50 is also applied as the base encoder network for comparison purposes, which is explained in detail in the experimental sections.

### 3.3. Projection Head

The purpose of using the projection head is to map the representations generated by the base encoder network to a 128-dimensional latent space, which serves as the basis for classification. Usually, the projection head can be a single linear layer network or an MLP with only one hidden layer. In this research, to further reduce the computational complexity and reflect the advantage of the proposed framework, the linear layer network is used as the projection head by default. The option of using an MLP with one hidden layer will be considered in future work.

### 3.4. Contrastive Loss Function

The contrastive loss function for self-supervised learning used in this paper is the Normalized Temperature-scaled Cross-Entropy Loss (NT-Xent Loss) [18] (Equation (1)):

(1) $$L^{self}=-\sum_{i\in I}\log\frac{\exp(z_{i}•z_{j(i)}/\tau )}{\sum_{a\in A(i)}\exp(z_{i}•z_{a}/\tau )}$$

where i∈I≡{1,2,…,2N} is the index of one arbitrary augmented sample, which is called the anchor. *N* refers to the size of mini-batch. $z_{l}=PROJ\left(ENC\left({\tilde{x}}_{l}\right)\right)\in {\mathbb{R}}^{D_{P}}$ is the 128-dimensional latent space obtained from the augmented sample ${\tilde{x}}_{l}$, which is learned by the base encoder network followed by the projection network. $z_{j\left(i\right)}$ refers to the augmented sample generated by the sample with index *i*. *j(i)* is called the positive, and the remaining 2(*N* − 1) indices are called the negatives. The symbol • denotes the inner product. $\tau \in {\mathbb{R}}^{+}$ is a temperature parameter. A(i)≡I\{i} represents the index value of all values of set *I* in 2*N* except *i*.

Triplet loss [16] is a special case of the loss defined in SupCon [14], with only one positive and one negative sample. The margin can be expressed as two times of temperature in the loss function defined in the supervised contrastive learning. For N-pair [17], although the loss does not define a temperature parameter, it can be considered as a case of SupCon loss with a certain transformation. As for SimCLR [18], when the all-positives set in the 2*N* samples is restricted to contain only a view of the same source action as that of the anchor, the self-supervised contrastive loss can be expressed in a form of supervised contrastive learning [14]. The above conclusions can be mathematically proved [14].

Therefore, the loss function definition of SupCon can not only be a self-supervised learning loss but also be used as supervised contrastive learning with human-annotated labels. When the supervised contrastive loss function is employed, the augmented sample and samples with the same label in a mini-batch are all simultaneously used as positives. Moreover, the gradient calculation of SupCon has the intrinsic ability to mine hard positives and negatives, which ensures the maximum utilization of hard samples [14].

The supervised contrastive learning loss function adopted in this paper is a unity of the above contrastive losses (Equation (2)) [14]:

(2) $$L^{\sup}=\sum_{i\in I}\frac{-1}{\left|P(i)\right|}\sum_{p\in P(i)}\log\frac{\exp\left(z_{i}•z_{p}/\tau \right)}{\sum_{a\in A(i)}\exp(z_{i}•z_{a}/\tau )}$$

where $P\left(i\right)\equiv \left\{p\in A\left(i\right):{\tilde{y}}_{p}={\tilde{y}}_{i}\right\}$ corresponds to the set of indices of all positives in *A(i)* except *i*. |P(i)| is its cardinality.

### 3.5. Classifier

The classifier adopts a linear classification network with one single, fully connected layer followed by a cross-entropy as the loss function.

### 3.6. The Superiority of FLOPs

The default base encoder network used in this paper is an MLP with 256 hidden layers. The FLOPs of the MLP network are the summation of the FLOPs of the encoder and the head. The fully connected layer in the encoder contributes most of the portion, while the computation of the head is very low. Meanwhile, using the ResNet50 as the base encoder network is an option in the experimental sections for comparison purposes. Because the ResNet50 network has relatively larger FLOPs, only the FLOPs of the encoder are considered, while the FLOPs of the head can be ignored. To reflect the strength of ConMLP in computational complexity, the FLOPs of typical GCN models are also listed as a reference.

Table 1 shows an evaluation of Shift-GCN [34], ST-GCN [6], 2s AS-GCN [46], 2s AGCN [31], 2s AGC-LSTM [47], and 4s DGNN [48]. Moreover, prefix 2s refers to a two-stream fusion strategy by using “joint stream” and “bone stream”, and 4s refers to the addition of “joint motion stream” and “bone motion stream” [34]. The GCN-based models generally require large FLOPs, which are several orders of magnitude larger than ConMLP. Although the computational complexity of ConMLP is relatively low, the performance of ConMLP can be comparable to that of GCN-based state-of-the-art models.

The computational complexity of MLP- and ResNet50-based networks are all calculated by an openly available FLOPs computing framework [49]. The FLOPs of the GCN-based model are from the analysis with Shift-GCN [34]. All the FLOPs in this section are based on training one single action sample.

## 4. Experiments

### 4.1. Datasets

#### 4.1.1. NTU RGB+D Datasets

NTU RGB+D [50] contains 56,880 action clips in 60 classes, which were simultaneously captured by 3 camera views. The recommendation metric protocols are Cross-View (X-View) and Cross-Subject (X-Sub). NTU RGB+D 120 [51] is an expansion of NTU RGB+D. It contains 114,480 clips in 120 classes. Similarly, Cross-Setup (X-Set) and Cross-Subject (X-Sub) are recommended as evaluation protocols.

#### 4.1.2. Data Extraction and Pre-Processing

In this research, the raw data were divided into a training set and a test set. According to the official NTU dataset statement, there are some incomplete data in the datasets, which needed to be removed accordingly. The training and test sets used in this paper were as follows: For NTU RGB+D, there were 37,646 and 18,932 clips for X-View, while there were 40,091 and 16,487 clips for X-Sub. For NTU RGB+D 120, there were 54,468 and 59,477 clips for X-Set, while there were 63,026 and 50,919 clips for X-Sub, respectively.

### 4.2. Default Metrics

MLP was applied as the base encoder network, which was optimized by SGD with a learning rate of 0.001 and weight decay of 0.0005. Additionally, a linear project head was used to transform the representations to a 128-dimensional latent space. The same definition of supervised contrastive learning [14] was chosen as the loss function, with a temperature of 0.07. A mini-batch size of 512 for training was randomly generated for 5000 epochs. The learning rate was decayed with a cosine schedule without restarts [52].

### 4.3. Comparison with the State of the Art

ConMLP was evaluated on two datasets, NTU RGB+D [50] and NTU RGB+D 120 [51]. Compared with other self-supervised approaches, ConMLP achieved the state of the art on several views of the datasets. It achieved the highest recognition rate on the X-View of NTU RGB+D, with reduced performance on all three other views. The results of the other methods are similar to this. Overall, the performances of these methods on NTU RGB+D 120 are generally not as good as those on NTU RGB+D due to the increased number of classes (Table 2).

On the other hand, in terms of supervised learning methods, the performance of ConMLP is also very outstanding. Similar to the results of self-supervised learning, the accuracies on certain views achieve the state of the art (Table 3).

To verify whether the MLP-based encoder network was more advantageous under the lower FLOPs demand, a ResNet50-based encoder network with a nonlinear neural network as the project head was also evaluated under the same evaluation metrics. The experimental results demonstrated that the MLP-based encoder network with 256 hidden layers has higher overall performance (Table 2 and Table 3).

### 4.4. The Case without Contrastive Learning

To validate the superiority of the contrastive learning method, a model with no contrastive learning loss function setups but a cross-entropy loss function, was evaluated for comparison purposes. Each individual skeleton sequence was fed directly into the base encoder network without any data augmentations. The representations from the project head were classified by the cross-entropy loss function instead of the contrastive loss function. Overall, the accuracies of the model with contrastive learning loss function were better. Moreover, the results obtained by self-supervised contrastive learning even surpassed the case in which supervised cross-entropy loss was deployed (Table 4).

### 4.5. Determine the Optimal Hyperparameters

As for the default settings, a learning rate of 0.001, temperature of 0.07, and epochs of 5000 were applied. The above optimal combination of hyperparameters is based on a series of experiments.

The learning rate was checked for values of 0.0001, 0.001, 0.005, 0.01, and 0.1. The bigger the learning rate, the worse the performance. However, when the learning rate was adjusted to 0.0001, the recognition accuracy dropped dramatically. The best accuracy was achieved when the learning rate was 0.001 (Table 5).

Temperature is considered a critical factor for contrastive learning. The lower the temperature, the greater the contribution to penalizing the hard cases. However, contrastive loss concentrates a few nearest samples when a very low temperature is applied, which can seriously degenerate the performance [53]. Additionally, five values were evaluated: 0.01, 0.04, 0.07, 0.1, and 0.5. The best recognition accuracy was achieved at the temperature of 0.07 (Table 6). This is also consistent with the default setting in SupCon [14]. These results demonstrate that an extremely low temperature can cause an obvious decrease in recognition accuracy.

The number of epochs explored was high. In the cases in which the epochs of 1000, 2000, 3000, 4000 and 5000 were measured, our results show that the larger the epoch, the better the recognition performance (Table 7). In addition, there was no significant performance improvement after taking the epoch value above 5000.

Moreover, an ablation study for cosine schedule [52] was adopted, and no significant influence was observed.

### 4.6. Number of Hidden Layers Is Critical

Another reason contributing to the excellent results of the MLP architecture is the number of hidden layers. An MLP with 256 hidden layers was employed as the base encoder network by default in this paper.

To verify the reason why the MLP architecture outperforms its ResNet50 counterpart, the number of hidden layers of the MLP was gradually decreased from 256 to 128, 64, 32, and 16. However, as the number of hidden layers decreased, the performance decreased dramatically (Table 8). This also confirms that more network layers lead to more useful features being learned.

As for deeper network architecture, skip connections [43] can be set [36,37,40]. This will be explored in future work.

### 4.7. Computational Complexity and Numbers of Parameter

In this section, the computational complexity and the number of parameters of both the MLP-based model and the ResNet50-based model are compared and analyzed. The FLOPs and the number of parameters listed in Table 9 are the values when processing one action sample. Because the model needs to be utilized in training and inference, it should be calculated twice, whereas the classifier is only utilized at inference time.

Although the parameters of the ResNet50 model are not much larger than those of the MLP model, the difference in computational cost is several orders of magnitude. Therefore, models using MLP as the base network have considerable advantages in terms of computational resource consumption. Nevertheless, the recognition accuracy of the MLP-based contrastive learning framework still achieves state-of-the-art performance.

Moreover, compared with the GCN-based architecture, even Shift-GCN [34], which is known for its low computational complexity, in addition to ConMLP, still has advantages. Shift-GCN achieves an accuracy of 95.1% on NTU RGB+D; yet it claimed as many as 2.5G FLOPs. In contrast, ConMLP’s FLOPs were only 46.54M, but with an accuracy of 96.9%.

## 5. Conclusions

The aim of this research is to recognize human actions through skeleton data. The goal is to control the consumption of computing resources and reduce the computational complexity of the model as much as possible while keeping the high recognition accuracy.

ConMLP, which is proposed in this paper, is a simple learning framework based on a naive MLP architecture. In this paper, the performance of the model on NTU RGB+D and NTU RGB+D 120 datasets while using self-supervised contrastive learning is mainly analyzed. Additionally, the loss function is extended to the case of supervised learning. The corresponding model performances are analyzed in comparison with both self-supervised learning and supervised learning approaches.

Moreover, the ResNet50 is used as an alternative option to the base encoder network to make a comparison with the MLP-based network. On the premise of obtaining state-of-the-art recognition accuracies, the advantages of ConMLP, such as low computational complexity and a small number of parameters, are highlighted.

## Figures and Tables

**Table 1:** Computational complexity comparison between ConMLP- and GCN-based models.

Models	FLOPs (G)
4s DGNN [48]	126.80
2s AGC-LSTM [47]	54.40
2s AGCN [31]	35.80
2s AS-GCN [46]	27.00
ST-GCN [6]	16.20
Shift-GCN [34]	2.50
ConMLP *	1.30
ConMLP	0.05

Note that all the FLOPs are for processing one action sample. * Denotes using ResNet50 as the base encoder network.

**Table 2:** Top 1 accuracy comparison with self-supervised methods.

Models	NTU RGB+D		NTU RGB+D 120	
	X-View (%)	X-Sub (%)	X-Set (%)	X-Sub (%)
4s-MG-AL [20]	68.0	64.7	49.5	46.2
CRRL [21]	73.8	67.6	57.0	56.2
Tanfous et al. [22]	76.3	67.0	59.1	61.5
SKT [23]	77.1	72.6	64.3	62.6
GLTA-GCN [24]	81.2	61.2	51.1	49.1
MCAE-MP [27]	82.4	51.9	46.1	42.3
SRCL [19]	82.5	76.7	67.5	67.1
Thoker et al. [26]	85.2	76.3	67.9	67.1
Wang et al. [25]	85.8	80.2	85.5	84.2
VaRe [8]	96.7	92.0	89.4	87.6
ConMLP *	92.2	64.8	62.5	73.9
ConMLP	96.9	75.4	77.2	76.4

* Denotes using ResNet50 as the base encoder network.

**Table 3:** Top 1 accuracy comparison with supervised methods.

Models	NTU RGB+D		NTU RGB+D 120	
	X-View (%)	X-Sub (%)	X-Set (%)	X-Sub (%)
ST-GCN [6]	88.3	81.5	/	/
AS-GCN [46]	94.2	86.8	/	/
AGC-LSTM [47]	95.0	89.2	/	/
2s-AGCN [31]	95.1	88.5	/	/
DGNN [48]	96.1	89.9	/	/
MS-G3D [7]	96.2	91.5	88.4	86.9
Shift-GCN [34]	96.5	90.7	87.6	85.9
MST-GCN [33]	96.6	91.5	88.8	87.5
STF [9]	96.9	92.5	89.9	88.9
ConMLP *	93.0	73.9	75.7	87.5
ConMLP	96.9	75.1	87.2	77.8

* Denotes using ResNet50 as the base encoder network.

**Table 4:** Top 1 accuracy comparison with cross-entropy loss.

	Base Encoder Network	NTU RGB+D		NTU RGB+D 120	
		X-View (%)	X-Sub (%)	X-Set (%)	X-Sub (%)
SL with cross-entropy loss	ResNet50	91.4	64.4	75.4	75.3
	MLP	94.5	63.2	76.2	86.5
SSL with contrastive loss	ResNet50	92.2	64.8	62.5	73.9
	MLP	96.9	75.4	77.2	76.4
SL with contrastive loss	ResNet50	93.0	73.9	75.7	87.5
	MLP	96.9	75.1	87.2	77.8

SL and SSL denote supervised learning and self-supervised learning, respectively.

**Table 5:** Top 1 accuracy from various learning rates.

Dataset	Learning Rate				
	0.0001	0.001	0.005	0.01	0.1
NTU RGB+D X-View (%)	28.1	96.9	95.3	90.6	88.3

**Table 6:** Top 1 accuracy from various temperatures.

Dataset	Temperature				
	0.01	0.04	0.07	0.10	0.50
NTU RGB+D X-View (%)	89.8	95.3	96.9	95.3	92.2

**Table 7:** Top 1 accuracy from various epochs.

Dataset	Epoch				
	1000	2000	3000	4000	5000
NTU RGB+D X-View (%)	85.2	93.8	96.1	96.1	96.9

**Table 8:** Top 1 accuracy from various numbers of hidden layers.

Dataset	Number of Hidden Layers				
	16	32	64	128	256
NTU RGB+D X-View (%)	13.3	17.2	71.1	93.0	96.9

**Table 9:** Computational complexity and parameters comparison.

		FLOPs (M)	Parameters (M)
	Model	23.24 × 2	11.62 × 2
MLP-based	Classifier	0.06	0.03
	Total	46.54	23.27
	Model	655.32 × 2	23.67 × 2
ResNet50-based	Classifier	0.50	0.25
	Total	1311.14	47.59

Note that all the FLOPs are for processing one action sample.

## Data Availability

The data presented in this study are openly available in NTURGB-D at https://doi.org/10.1109/CVPR.2016.115 and https://doi.org/10.1109/TPAMI.2019.2916873, reference number [50,51].
